# Tormenting thoughts: The posterior cingulate sulcus of the default mode network regulates valence of thoughts and activity in the brain's pain network during music listening

**DOI:** 10.1002/hbm.25686

**Published:** 2021-10-15

**Authors:** Stefan Koelsch, Jessica R. Andrews‐Hanna, Stavros Skouras

**Affiliations:** ^1^ Department of Biological and Medical Psychology University of Bergen Bergen; ^2^ Department of Psychology, Cognitive Science University of Arizona Tucson Arizona USA

**Keywords:** brain, emotion, mind‐wandering, default mode network

## Abstract

Many individuals spend a significant amount of their time “mind‐wandering”. Mind‐wandering often includes spontaneous, nonintentional thought, and a neural correlate of this kind of thought is the default mode network (DMN). Thoughts during mind‐wandering can have positive or negative valence, but only little is known about the neural correlates of positive or negative thoughts. We used resting‐state functional magnetic resonance imaging (fMRI) and music to evoke mind‐wandering in *n* = 33 participants, with positive‐sounding music eliciting thoughts with more positive valence and negative‐sounding music eliciting thoughts with more negative valence. Applying purely data‐driven analysis methods, we show that medial orbitofrontal cortex (mOFC, part of the ventromedial prefrontal cortex) and the posterior cingulate sulcus (likely area 23c of the posterior cingulate cortex), two sub‐regions of the DMN, modulate the valence of thought‐contents during mind‐wandering. In addition, across two independent experiments, we observed that the posterior cingulate sulcus, a region involved in pain, shows valence‐specific functional connectivity with core regions of the brain's putative pain network. Our results suggest that two DMN regions (mOFC and posterior cingulate sulcus) support the formation of negative spontaneous, nonintentional thoughts, and that the interplay between these structures with regions of the putative pain network forms a neural mechanism by which thoughts can become painful.

## Graphical abstract

We used resting‐state functional magnetic resonance imaging (fMRI) and music to evoke mind‐wandering, with positive‐sounding music eliciting thoughts with more positive valence and negative‐sounding music eliciting thoughts with more negative valence. We observed that medial orbitofrontal cortex (mOFC) and the posterior cingulate sulcus, two sub‐regions of the default mode network (DMN), modulate the valence of thought‐contents during mind‐wandering, and that the posterior cingulate sulcus, a region involved in pain, shows valence‐specific functional connectivity with core regions of the brain's putative pain network. Results indicate that mOFC and the posterior cingulate sulcus support the formation of negative mind‐wandering thoughts, and that the interplay between these structures with regions of the putative pain network forms a neural mechanism by which thoughts can become painful.

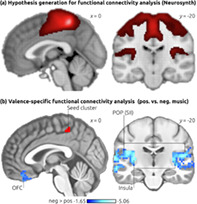

## 1 INTRODUCTION

The default mode network (DMN, or just default network, DN) is a brain network that becomes engaged during neuroimaging experiments when individuals are “left to think to themselves undisturbed” (Andrews‐Hanna, Reidler, Sepulcre, Poulin, & Buckner, 2010), that is, when people are not engaged in external interactions and their “minds wander” (Buckner, Andrews‐Hanna, & Schacter, 2008). Such spontaneous, nonintentional thoughts can be positive, such as daydreaming about positive things, or negative, such as ruminating on emotionally distressing issues. While there is disagreement on how to define “mind‐wandering” (Christoff et al., 2018; Christoff, Irving, Fox, Spreng, & Andrews‐Hanna, 2016; Seli et al., 2018) there is general consensus (i) that spontaneous, nonintentional thoughts can be positive or negative, (ii) that many individuals spend a significant amount of their time mind‐wandering, and (iii) that a significant portion (about 10–30%) of mind‐wandering is associated with negative thoughts (Fox et al., 2018; Killingsworth & Gilbert, 2010). Importantly, mind‐wandering with negative thought content is often associated with negative moods: mind‐wandering with negative thoughts content can cause negative mood (Killingsworth & Gilbert, 2010; Poerio, Totterdell, & Miles, 2013; Ruby, Smallwood, Engen, & Singer, 2013) and negative mood can cause mind‐wandering with negative thought content (Smallwood, Fitzgerald, Miles, & Phillips, 2009; Smallwood & O'Connor, 2011).

Surprisingly little is known about the neural basis of positive or negative spontaneous, nonintentional thoughts, and to the best of our knowledge only very few functional neuroimaging experiments modulated the valence of thoughts to specify DMN activity associated with positive or negative thoughts during mind‐wandering. A study by Tusche, Smallwood, Bernhardt, and Singer (2014) used a task with either positive or negative self‐referential attribution, and multivariate pattern analysis (MVPA) to identify voxels providing significant classification information for positive versus negative thoughts. Such voxels were found in the medial orbitofrontal cortex (mOFC, a part of the DMN). Notably, that classifier predicted the valence of thoughts during task‐free rest periods acquired during a second fMRI experiment 1 week later. Another study (Gorgolewski et al., 2014) assessed self‐generated thoughts during fMRI with a questionnaire. Activity in the fronto‐median cortex (extending into the mOFC) was negatively correlated with the questionnaire‐factor “positive thoughts.” A study by Taruffi, Pehrs, Skouras, and Koelsch (2017) used sad‐sounding and joyful‐sounding music to modulate the quality of thoughts during mind‐wandering. Functional neuroimaging data showed regions with higher eigenvector centrality (i.e., regions with influential positions within brain networks) during sad‐sounding than joyful‐sounding music in the ventromedial prefrontal cortex (vmPFC) including medial orbitofrontal cortex, and the anterior portion of the posterior cingulate cortex (area 23c of the PCC). Both mOFC and area 23c are subregions of the anterior and posterior midline “hubs” of the DMN (Andrews‐Hanna et al., 2010). Other studies support the notion that DMN activity modulates the valence of thoughts during mind‐wandering. A study by Karapanagiotidis et al. (2020) used resting‐state fMRI data and retrospective descriptions of the participants' experience during the scan. The authors reported that a state associated with more negative thoughts related to an individual's past, was associated with DMN activity. In a study by Letzen and Robinson (2017), individuals were asked to experience the emotions of positive (“happy”) and negative (“sad”) autobiographical memories. The authors observed that pre‐defined midline DMN regions showed increased functional connectivity with ventral anterior cingulate cortex (vACC) and posterior insula during negative thoughts compared to baseline. Taken together, these studies suggest a role of anterior and posterior midline structures of the DMN (including mOFC and area 23c) in generating and driving thoughts with emotional valence during mind‐wandering, and that these DMN structures show functional connectivity with other structures, such as vACC and posterior insula, as a function of the valence of self‐generated thoughts.

To further investigate this issue, we developed an experimental paradigm in which spontaneous, nonintentional thoughts are stimulated and modulated with music. In two previous studies (Koelsch, Bashevkin, Kristensen, Tvedt, & Jentschke, 2019; Taruffi et al., 2017), we showed that music, in addition to influencing emotions and moods, also often elicits mind‐wandering, and modulates the valence of thoughts during mind‐wandering. In the study by Taruffi et al. (2017), thoughts emerging during joyful‐sounding music were more positive than thoughts emerging during sad‐sounding music. These results were replicated in a second study (Koelsch et al., 2019), in which heroic‐sounding music led to more positive thought contents than sad‐sounding music. However, the study by Taruffi et al. (2017) left a number of controversial questions open: First, the strength of mind‐wandering was higher during sad than joyful music, possibly due to sad music being slower and thus attracting less attention than fast music. Thus, it is unclear whether increased eigenvector centrality observed in the mOFC and area 23c of the posterior cingulate cortex modulated the valence of thought contents, or reflected the strength of mind‐wandering. Second, due to the lack of a neutral baseline condition, it was not possible to determine whether thought contents during sad music were actually negative, or just less positive than during joyful music. Nevertheless, that study (Taruffi et al., 2017) was an ideal basis for the formulation of directional hypotheses for our current study.

Here, we devised an experimental paradigm with two types of positive music stimuli (joyful and peaceful) and two types of negative music stimuli (sad and nervous) in a way that positive music had on average the same tempo as negative music (joyful and nervous music was faster than peaceful and sad music). The aim, thus, was to modulate the valence of thought contents while controlling for the tempo of the music (and thus for the strength of mind‐wandering), in order to identify differences in DMN activity between positive and negative thought. Moreover, we included rest‐blocks without music, to compare data of blocks with positive or negative music to a neutral baseline condition. Based on our previous studies (Koelsch et al., 2019; Taruffi et al., 2017) we hypothesized that music would evoke mind‐wandering with more positive thoughts during positive music, and more negative thoughts during negative music (compared to a neutral baseline), and stronger network (eigenvector) centrality in the midline‐core of the DMN during negative compared with positive music (and compared with a neutral baseline), specifically in the medial orbitofrontal cortex and area 23c of the posterior cingulate cortex (for an illustration of eigenvector centrality see Figure S1). Should our findings accord with our hypothesis, they would indicate a neural basis for the modulation of the valence of thoughts during mind‐wandering.

## 2 METHODS

### 2.1 Participants

Thirty three participants (18 women, age‐range 20–31 years, mean = 23.1 years) were included in the fMRI data analysis. Exclusion criteria were hearing impairment (as assessed with a standard pure‐tone audiometry), musical anhedonia (as assessed with the Barcelona Music Reward Questionnaire; Mas‐Herrero, Marco‐Pallares, Lorenzo‐Seva, Zatorre, & Rodriguez‐Fornells, 2013), and previously diagnosed neurological or psychiatric disorder (according to self‐report). None of the participants were professional musicians, 11 participants had never played an instrument, nine participants played an instrument or sang, but without having received instrument or singing lessons, and nine participants had received instrumental or singing lessons for an average of 3.5 years. Each participant provided written informed consent, and the study was approved by the regional ethics committee (Regional Ethics Committee of Western Norway, REK, ID 7332).

### 2.2 Stimuli

Stimuli were carefully pre‐selected based on pilot‐data to induce four target emotions: “joy”, “sad”, “peaceful”, and “nervous” (all of which are typically elicited by music in Western listeners; Zentner, Grandjean, & Scherer, 2008). For each of these emotions, five instrumental music stimuli (without lyrics) were chosen from the following genres: Rock (2 stimuli per emotion), Orchestral in film‐music style (2 stimuli per emotion), and Classical‐symphonic (1 stimulus per emotion), resulting in 20 stimuli (5 per emotion). The detailed list of stimuli is provided in Data S2. Each of these stimuli was cut such that the duration was about 1 min (0:58–1:09 min), then edited to have 1.5 s fade in/out ramps, and finally normalized (root mean square amplitude). Within each musical genre, all emotion stimuli had comparable instrumentation, thus balancing the overall timbre of stimuli across emotion categories. Moreover, *joy* and *nervous* stimuli were carefully matched to have the same average tempo (as measured in beats per minute, BPM). Likewise, *peaceful* and *sad* stimuli had the same average BPM. Thus, *positive* (joy, peaceful) and *negative* (nervous, sad) music stimuli had on average the same musical tempo, such that physiological arousal due to beat induction would not differ between positive and negative stimuli.

### 2.3 Preparatory internet experiment

**FIGURE 1:**
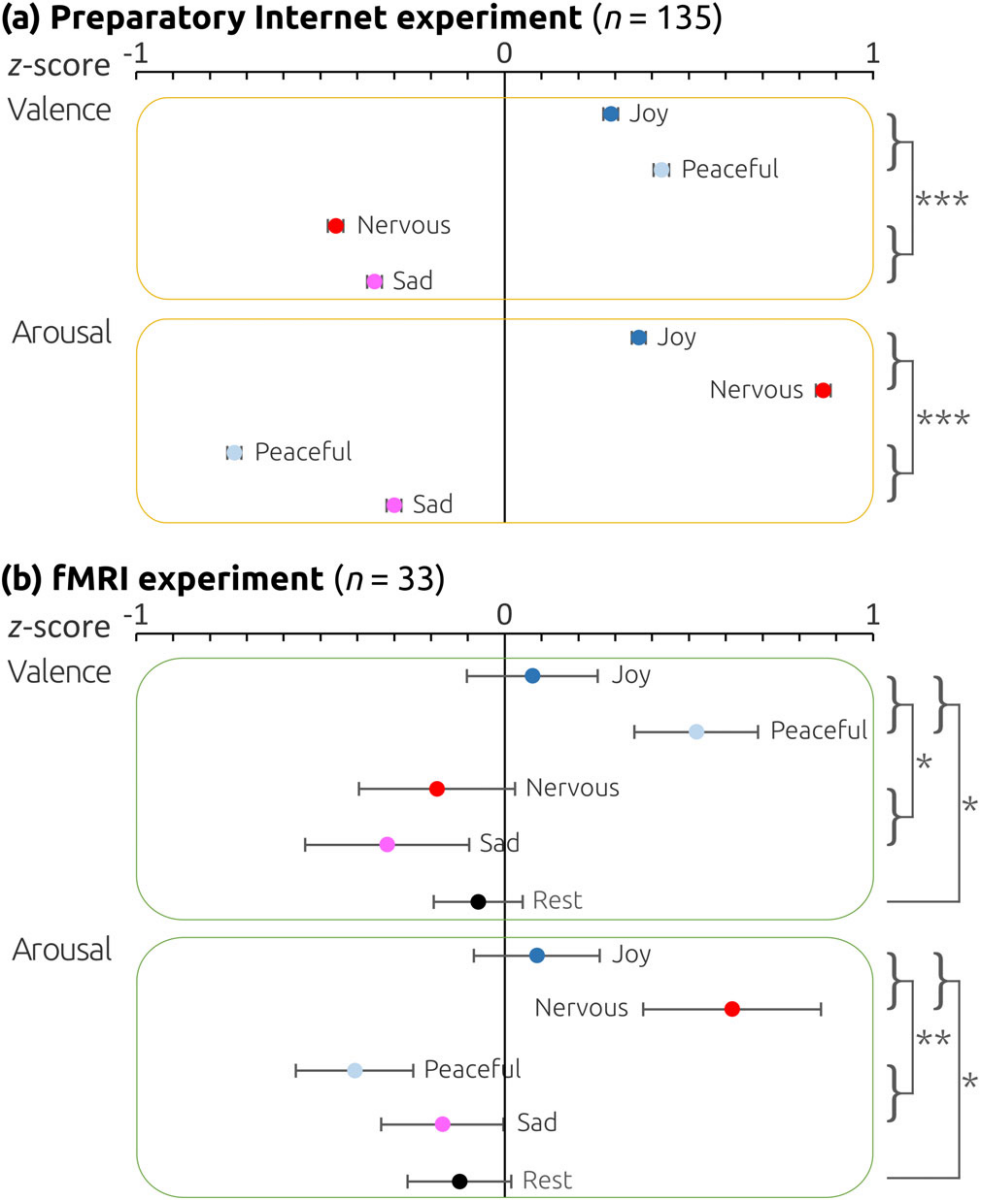
Valence and arousal of thought contents during mind‐wandering. The yellow‐framed boxes indicate results of the preparatory internet experiment (a), green‐framed boxes indicate results of the fMRI experiment (b). Blue disks represent positive‐sounding music (joy, peaceful), red disks negative‐sounding music (nervous, sad), dark disk colors indicate fast music (joy, nervous), and light disk colors indicate slow music (peaceful, sad). Black disks indicate mean ratings of rest blocks in the fMRI experiment (no rest blocks were administered in the internet experiment). Data were *z*‐transformed (separately for the preparatory internet experiment and the fMRI experiment). In both experiments, the valence of thoughts was higher for positive than negative‐sounding music, and the arousal of thoughts was higher for fast than for slow music. *Note*: ***p < .001; **p < .01; *p < .05

To characterize stimuli with regard to their potential to elicit mind‐wandering and influence the quality of thoughts, stimuli were evaluated in an internet experiment with *n* = 135 included participants, each completing two trials per emotion (joy, peaceful, nervous, sad). Thus, across all participants, 270 valid trials were gathered for each emotion. Participants listened to the music stimuli with the instruction to close their eyes and relax. After each stimulus, thought probes were collected, consisting of 10 questions. These questions were identical to a previous study (Koelsch et al., 2019) and are listed in Data S3. The questions assessed the control of the focus of attention, intensity of mind wandering, level of meta‐awareness, affective valence of thoughts, arousal of thought content, self‐referentiality, reference to other people, time relation, as well as relevance of thought contents to current concerns of the participant's life. Mind‐wandering was assessed with the answer “yes/no” in response to the item “Were your thoughts completely focused on the music?”, and occurred with a similar frequency across all four music conditions (during joyful stimuli in 198 out of the 270 trials, during peaceful stimuli in 210, during nervous stimuli in 195, and during sad stimuli in 203 trials). Importantly, the valence of thoughts (as assessed by the item “Was what you were thinking about rather negative or positive?”) was influenced by the valence of the music, such that positive music evoked thoughts with more positive valence, and negative music evoked thoughts with more negative valence (*F*[1,103] = 76.91, *p* < .00001, *η* ^2^ = .43, observed power: 1.0; see Figure 1a and Data S4 for details). Moreover, the arousal of thought contents (as assessed with the item “Was what you were thinking about making you rather calm or agitated?”) was higher during fast compared to slow music (*F*[1,103] = 249.88, *p* < .00001, *η* ^2^ = 0.71, observed power: 1.0; see Figure 1a for details), and higher during negative compared to positive music (*F*[1,103] = 44.17, *p* < .00001, *η* ^2^ = .30, observed power: 1.0). No other effects nor any interactions were observed for these variables, nor for any of the other collected items. Thus, our selected stimulus material had a strong effect on the valence and arousal of thoughts.

### 2.4 fMRI session: Procedure

In the fMRI experiment, music stimuli were concatenated (separately for each emotion condition) and presented in blocks, resulting in four music blocks (joy, peaceful, nervous, sad). In addition, the experimental paradigm included two rest blocks. Each block had a duration of 5 min. Ordering of the six blocks was pseudo‐randomized (such that the second rest block would never follow directly the first rest block) and counterbalanced across participants. The task for the participants was to relax (in both the rest and the music blocks), and to listen to the music (in the music blocks). At the beginning of each block, participants were asked to keep their eyes closed until the end of the block, when a beep tone signaled to open the eyes and commence the rating procedure. Then, 10 questions were presented visually, and participants responded to each question on a six‐point scale via two fMRI‐compatible response buttons (one in each hand), using their index fingers. The direction of the scales was counterbalanced across trials, to exclude potential motor preparation during the music blocks and thus potential response bias. The wording of the questions was identical to a previous study (Koelsch et al., 2019) and the preparatory internet experiment (Data S3). The rating procedure was practiced before the fMRI session.

### 2.5 Apparatus

MRI acquisition was carried out with a 3.0 T MRI machine (General Electric, Chicago, IL) at the radiology department of the Haukeland University Hospital in Bergen, Norway. A 32‐channel phased‐array head coil was used. A three‐dimensional T1‐weighted, high‐resolution anatomical image was acquired for each participant using a magnetization‐prepared rapid acquisition gradient echo (MP‐RAGE) sequence (repetition time [TR] 2,250 ms, echo time [TE] 30 ms, voxel size 1 × 1 × 1 mm). A gradient echo, echo‐planar pulse sequence (1172 functional volumes; TR 2250 ms; TE 30 ms; 3 mm slice thickness; 37 axial, sequential, top‐down slices with a 0.6 mm gap; flip angle = 70°; matrix = 64 × 64; FOV = 192 × 192 mm) sensitive to BOLD contrast was used to acquire functional data. Additionally, the acquisition window was tilted by a 30° angle to the intercommissural plane (AC‐PC); this procedure is helpful in reducing the susceptibility to artifacts in areas such as the orbitofrontal cortex and temporal lobe (Weiskopf, Hutton, Josephs, Turner, & Deichmann, 2007). Visual stimuli were presented via an in‐room monitor, and audio‐stimuli were presented via MRI compatible headphones (model NNL HP‐1.3, NordicNeuroLab, Bergen, Norway) with a flat range response of 8–35,000 Hz and an external noise attenuation of an A‐weighted equivalent continuous sound level of 30 dB.

### 2.6 Behavioral data analysis

Behavioral data obtained in the preparatory internet survey and during the fMRI experiment were analyzed using SPSS 26 (IBM, Armonk, NY). Only data from 29 participants of the fMRI were analyzed because of data loss due to technical reasons.

### 2.7 Functional MRI data analysis

Datasets were preprocessed using standard methods implemented in SPM12 (Wellcome Trust Centre for Neuroimaging, London, UK; RRID:SCR_007037) and the MIT connectivity toolbox (Connectivity Toolbox; RRID:SCR_009550), consisting of slice‐time correction, realignment and reslicing of functional volumes, artifact detection (using the Artifact Detection Tools, RRID:SCR_005994), denoising via regression of average white matter timeseries, average CSF timeseries, 24 Volterra expansion movement parameters and scan‐nulling regressors (Lemieux, Salek‐Haddadi, Lund, Laufs, & Carmichael, 2007). Normalization to the sample's optimal template and from that to the ICBM MNI template (Fonov et al., 2011) was performed in a single step using SyGN (Tustison et al., 2013). Spatial smoothing using a 3D Gaussian kernel and a filter size of 6 mm at FWHM, as well as temporal high‐pass filtering with a cut‐off frequency of 1/90 Hz, were performed in a single step using the Leipzig Image Processing and Statistical Inference Algorithms (LIPSIA v2.2.7, RRID:SCR_009595). Voxel‐wise eigenvector centrality mapping (ECM), an advanced graph theory method accounting for whole‐brain functional connectomics (for illustration of eigenvector centrality see Data S1), was computed based on positive correlations, similarly as in previous studies (e.g., Koelsch & Skouras, 2014; Skouras et al., 2019; Taruffi et al., 2017), yielding ECM images for each condition and for each subject. (Note that both positive and negative conditions contained both arousing and relaxing stimuli, and that the overall level of arousal was balanced between positive and negative stimuli). ECM images were gaussianized voxel‐wise across all subjects (van Albada & Robinson, 2007), to enable second‐level parametric inference.

To compute the contrast between two music conditions (positive [joy and peaceful] vs negative [sad and nervous]), ECM images from all subjects were entered into a second‐level general linear model and a paired‐samples *t*‐test was performed, resulting in a *z*‐score map. Clusters with significant differences in eigenvector centrality between the two conditions were identified following correction for multiple comparisons by using a combination of single‐voxel probability thresholding as well as cluster‐size and cluster‐*z*‐value thresholding, through 10,000 iterations of Monte Carlo simulations. The initial cluster threshold of randomly generated maps of *z*‐values was set to a probability level equivalent to a one‐tail significance level of *p* = .01 (due to the replicatory nature of the study). The Monte Carlo simulations accounted for anatomical priors through hemispheric symmetry and generated thresholds for cluster sizes and peak *z*‐values based on the initial threshold and the specific geometrical properties of the images (i.e., number of voxels, voxel size, spatial extent and spatial smoothness) to control for false positives and obtain the final activation maps corrected for multiple comparisons at the *p* = .05 level of significance (Lohmann et al., 2010; van Albada & Robinson, 2007).

### 2.8 Valence‐specific functional connectivity analysis

The ECM results indicated a cluster in the posterior cingulate sulcus at the group level for the contrast negative versus positive music (replicating the finding of our previous study; Taruffi et al., 2017). This cluster was then used as a seed to compute its standardized functional connectivity, via Fisher's *r* to *z* transformation, with each gray matter voxel across the whole brain for each subject, for each condition (positive and negative) separately. On the second level, contrasts of positive and negative functional connectivity maps were computed via a paired‐samples *t*‐test, to obtain the *valence‐specific* (e.g., stronger during negative than positive) functional connectivity of the seed cluster. Clusters with significant differences between positive and negative stimuli, in relation to the seed cluster, were identified following correction for multiple comparisons, performed identically as for the ECM analysis, with the exception of setting the corrected level of significance at *p* = .001 (to protect against false positives).

## 3 RESULTS

### 3.1 Behavioral data

During the rest blocks, participants rated their *mind‐wandering* (conceptualized for this experiment as task‐unrelated thinking) with the item “How much were you thinking about the experiment versus something else?” on a scale from 1 to 6) on average as moderate (*M* = 4.1, *SD* = 1.4). During the music blocks, participants also reported moderate mind‐wandering (joy: *M* = 3.2, *SD* = 1.2; peaceful: *M* = 3.9, *SD* = 1.2; sad: *M* = 3.3, *SD* = 1.3; nervous: *M* = 3.2, *SD* = 1.0). Although mind‐wandering was nominally strongest during peaceful music, this difference (compared to the other music conditions) was not significant (corrected for multiple comparisons). Compared with rest, mind‐wandering was weaker during joy, sad, and nervous music (all *p* < .05, corrected for multiple comparisons), but not during peaceful music (*p* > .05). Importantly, replicating the results of our preparatory internet experiment (see Section 2), the *valence* of thought contents was significantly more positive during positive (joy, peaceful) than negative (nervous, sad) music (*F*[1,24] = 4.73, *p* = .04, *η* ^2^ = .17, observed power: .55; see Figure 1b and Data S5). One‐sided tests showed that, compared to rest, thought contents were significantly more positive during positive music (*F*[1,26] = 5.83, *p* < .05, *η* ^2^ = .18, observed power: .64), and tended to be more negative during negative music (*F*[1,26] = 1.77, *p* < .1). The *arousal* of thought contents was higher during fast (joy, nervous) than slow (peaceful, sad) music (*F*[1,26] = 8.99, *p* = .006, *η* ^2^ = .3, observed power: 0.82), but importantly (and contrary to the pilot experiment) there was no significant difference in the arousal of thought contents between *positive* and *negative* music (*p* = .07). Compared to rest, the arousal of thought contents was higher during fast music (*F*[1,26] = 3.95, *p* < 0.05 one‐sided, *η* ^*2*^ = .13, observed power: 0.48). No other main effects or interactions were observed for any of the other items. In summary, mind‐wandering emerged during all experimental blocks, the valence of thoughts was more positive during positive music, and the arousal of thought contents was higher during fast music (but did not differ between positive and negative music).

### 3.2 Eigenvector centrality mapping

**FIGURE 2:**
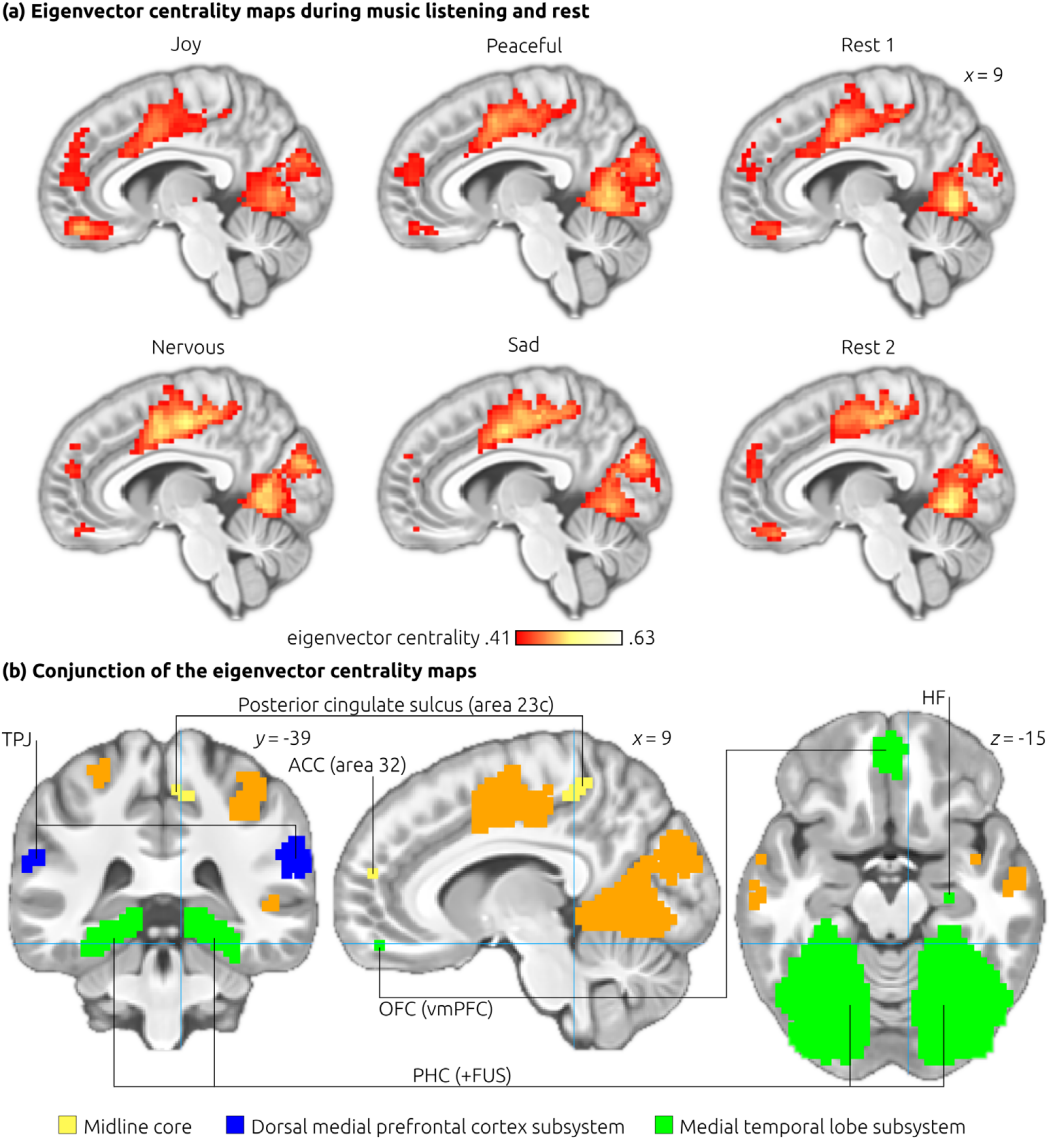
Eigenvector Centrality Mapping. (a) The eigenvector centrality maps (ECMs) for the six experimental blocks (ordering of stimulus and rest blocks was counterbalanced across subjects; rating procedures at the end of each block were not included in the analysis). The locations of centrality maxima elicited across all six blocks were remarkably similar. This similarity is demonstrated in the conjunction analysis of the six eigenvector centrality maps (b). Regions with high eigenvector centrality overlap were found in the “midline core” (yellow) of the default mode network (DMN), the “dorsal medial prefrontal cortex subsystem” (blue), and the “medial temporal lobe subsystem” (green, note that the PHC clusters extend outside the DMN into fusiform areas). Additional regions with high eigenvector centrality (orange) include the extrastriate medial occipital cortex, midcingulate cortex, lateral parietal lobe, and temporal cortex. Images are shown in neurological convention. The blue cross‐hairs indicate the coordinates of the slices shown. Only voxels with eigenvector centrality values ≥0.41 are shown, corresponding to the top tertile of centrality values (note that the centrality values do not indicate statistical significance). Coordinates refer to MNI space. ACC, anterior cingulate cortex; FUS, fusiform cortex; HF, hippocampal formation; OFC, orbitofrontal cortex; PCC, posterior cingulate cortex; PHC, parahippocampal cortex; TPJ, temporoparietal junction; vmPFC, ventro‐medial prefrontal cortex

Given that mind‐wandering emerged during all music conditions, we first investigated whether a similar eigenvector centrality pattern emerged during music and rest. We observed that the eigenvector centrality maps (ECMs) of the rest blocks showed a striking similarity with the ECMs of each of the music blocks (joy, peaceful, nervous, and sad): The ECM clusters strongly overlapped in all conditions (Figure 2a), with peak coordinates across all conditions located within the same or adjacent voxels. This remarkable overlap is demonstrated in the conjunction analysis of the ECMs across all conditions (Figure 2b): Any colored voxel in Figure 2b indicates that this voxel had a relatively high eigenvector centrality value in each of the six blocks (two rest blocks, and four music blocks). No other ECM‐clusters were observed in the two rest blocks, and no significant differences emerged in a direct contrast between any of the music blocks on the one hand, and the two rest blocks on the other (i.e., none of the music conditions differed significantly from the rest condition). These results indicate that the resting‐state network (as observed during rest) was also active during music (consistent with the observation that both rest and music conditions evoked mind‐wandering, as indicated by the behavioral data).

Notably, this resting‐state network, as assessed using ECM, is reminiscent of the Default Mode Network (DMN), as typically assessed using independent component analysis (ICA): Regions with high eigenvector centrality overlap with the “midline core” of the DMN (yellow in Figure 2b), with the “dorsal medial prefrontal cortex subsystem” of the DMN (blue in Figure 2b), and the “medial temporal lobe subsystem” of the DMN (green in Figure 2b). Additional regions with high eigenvector centrality observed in the present study (orange in Figure 2b) include the midcingulate cortex (MCC), extrastriate medial occipital cortex, lateral parietal lobe, and temporal cortex. Interestingly, as can be seen in Figure 2a, the areas with the highest eigenvector centrality values were not part of the DMN (in particular visual cortex, and MCC).

### 3.3 ECM contrasts

**FIGURE 3:**
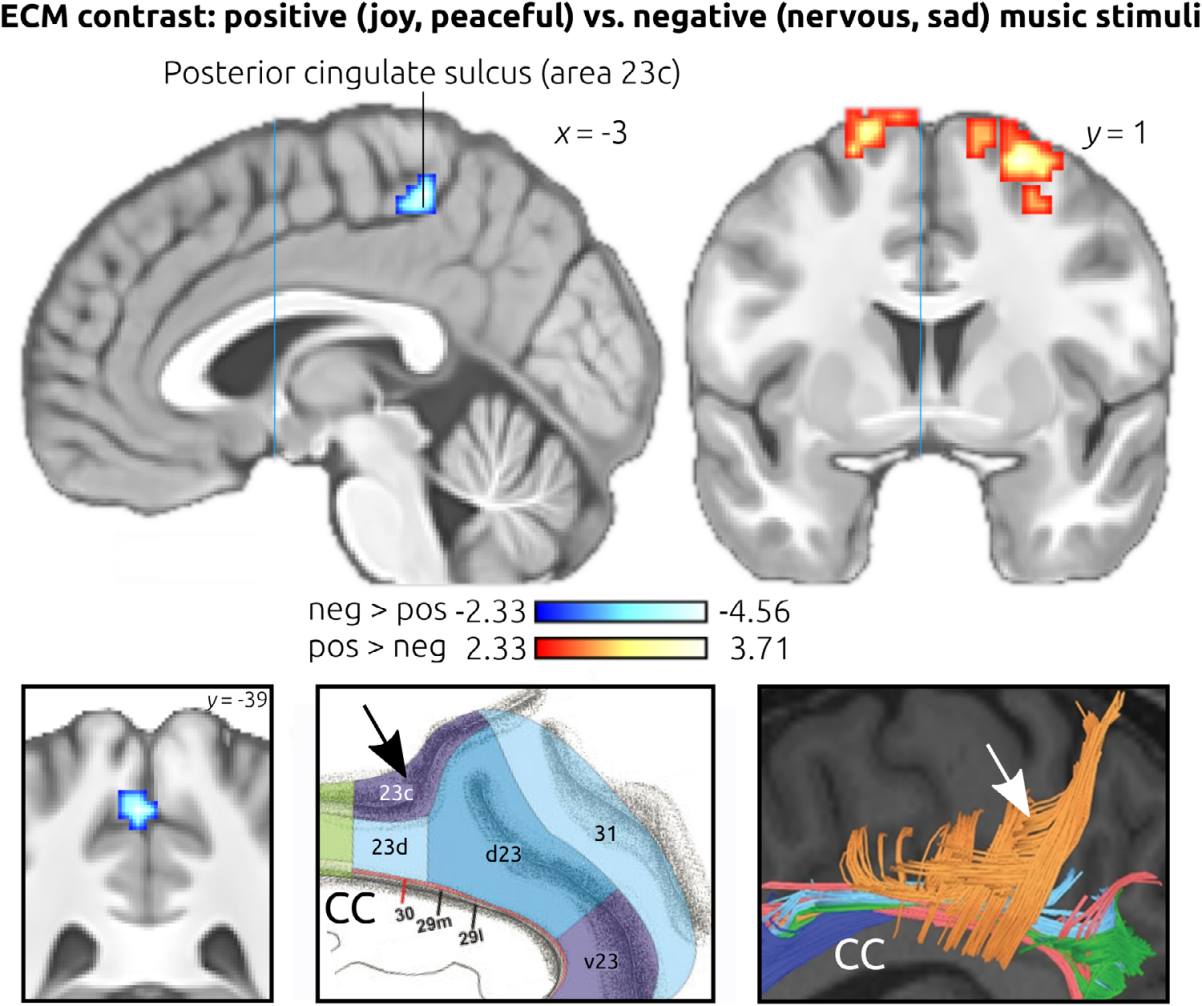
Contrast of eigenvector centrality maps. The top panel shows the ECM contrast of positive (joy & peaceful) versus negative (nervous & sad) music, corrected for multiple comparisons. The blue cluster indicates that, during negative‐sounding music (associated with negative valence of thought contents), higher network centrality emerged in the posterior cingulate sulcus (area 23c of the posterior cingulate cortex). During positive music (associated with positive thought contents) higher centrality emerged in the dorsolateral premotor cortex (yellow‐red clusters). Bottom panel: The left inset shows a coronal view of the cluster in area 23c, illustrating that the cluster includes the upper bank of the posterior cingulate sulcus. The middle inset shows the receptor‐architectonic parcellation of the posterior cingulate cortex according to Palomero‐Gallagher et al. (2009; the blue and violet colors indicate sub‐regions of the PCC). Note that area 23c covers both the lower and the upper banks of the cingulate sulcus. The black arrow indicates the approximate location of the ECM cluster in area 23c. The right inset shows white matter tracts of area 23c in orange, according to the connectomic atlas by Baker et al. (2018; the white arrow indicates the approximate location of the ECM cluster in area 23c). Images are shown in neurological convention, coordinates refer to MNI space, the color‐bars indicate *z*‐values. CC, corpus callosum

We then investigated differences in ECMs between positive (joy & peaceful) and negative (nervous & sad) music (note that the average tempo was identical for positive and negative music, leading to comparable arousal for positive and negative stimuli). Regions in which eigenvector centrality differed between positive and negative music are shown in Figure 3 (for details see Table 1; these regions were also revealed in an analogous analysis including age, sex, and framewise displacement as covariates, see Data S6). Higher centrality values during *negative* (compared to positive) music were indicated in the upper bank of the posterior cingulate sulcus, extending medially into the paracentral lobule (see also left inset in Figure 3). According to the four‐region model of the cingulate cortex proposed by Palomero‐Gallagher et al. (2009), this region likely corresponds to area 23c of the posterior cingulate cortex (PCC), which is located within, and in the close vicinity, of the posterior cingulate sulcus (see middle inset in Figure 3). The PCC thus extends into, and beyond, the upper bank of the cingulate sulcus, bordering anterior‐superiorly on area 5 (subfield 5 Ci). Likewise, a connectomic analysis of area 23c shows that white matter tracts of area 23c originate from within, and around, the posterior cingulate sulcus (see right inset in Figure 3). To the best of our knowledge, no anatomical probability map of area 23 is available. For example, this area has not been included yet in the SPM Anatomy toolbox (Eickhoff et al., 2005). Using the Anatomy toolbox (with its option “Overlap between structure and function”) we found that the probability for the cluster being located in area 5 is merely 19%, rendering it unlikely that neural activity in our cluster originated (only) from area 5.

**TABLE 1:** Contrast of eigenvector centrality maps (ECMs)

ECM contrast of positive vs. negative music stimuli				
Area	Vol. (mm^3^)	*z*‐value (mean ± SD)	*z*‐value (max)	MNI‐coord.
Post. cingulate sulcus (area 23c)	432	−2.70 ± 0.38	−3.72	−3, −39, 58
SFGp (BA6 left)	999	2.70 ± 0.34	3.74	−18, 0, 76
SFGp (BA6 right)	2,133	2.81 ± 0.50	4.56	24, 3, 67

*Note*: Results of the ECM contrast of positive (joy & peaceful) versus negative (nervous & sad) music. Negative *z*‐values indicate higher centrality during negative than positive stimuli (and vice versa). Results were corrected for multiple comparisons (*p* < .05), using 10,000 Monte Carlo permutations. BA: Brodmann area; SFGp: posterior superior frontal gyrus.

Compared with rest, centrality values of the cluster in the posterior cingulate sulcus were higher during negative music (max *z* = 2.87), and did not differ between rest and positive music. Thus, negative music increased the functional centrality of neurons in the posterior cingulate sulcus compared to both rest and positive music. Higher eigenvector centrality values during *positive* (compared to negative) music were indicated in the dorso‐lateral premotor cortex bilaterally (area 6). Contrary to our previous study (Taruffi et al., 2017), no significant differences between positive versus negative music were observed in the vmPFC.

### 3.4 Data‐driven hypothesis generation and functional connectivity analysis

The ECM contrast analysis revealed that the centrality of the posterior cingulate sulcus (area 23c of the PCC), varies depending on the valence of music‐evoked thoughts. To aid the interpretation of this finding, we (*first*) explored the functional significance of area 23c using meta‐analytic data, then (*second*) investigated the functional connectivity of this region, also using meta‐analytic data, and finally (*third*) computed the functional connectivity of area 23c using the fMRI data of the present study, comparing the functional connectivity of area 23c between positive and negative music in order to specify whether the functional connectivity of area 23c is modulated by emotions. These three steps are described in more detail in the following.

First, we identified the meta‐analytic activation pattern associated with the peak coordinate of the ECM cluster observed in area 23c (listed in Table 1) using the Neurosynth platform (neurosynth.org; Yarkoni, Poldrack, Nichols, Van Essen, & Wager, 2011). The top associated label (out of 525 labels), was “painful” (posterior *z* = 5.64), followed by the label “pain” (posterior *z* = 5.14). This indicates a role of area 23c in pain.

**FIGURE 4:**
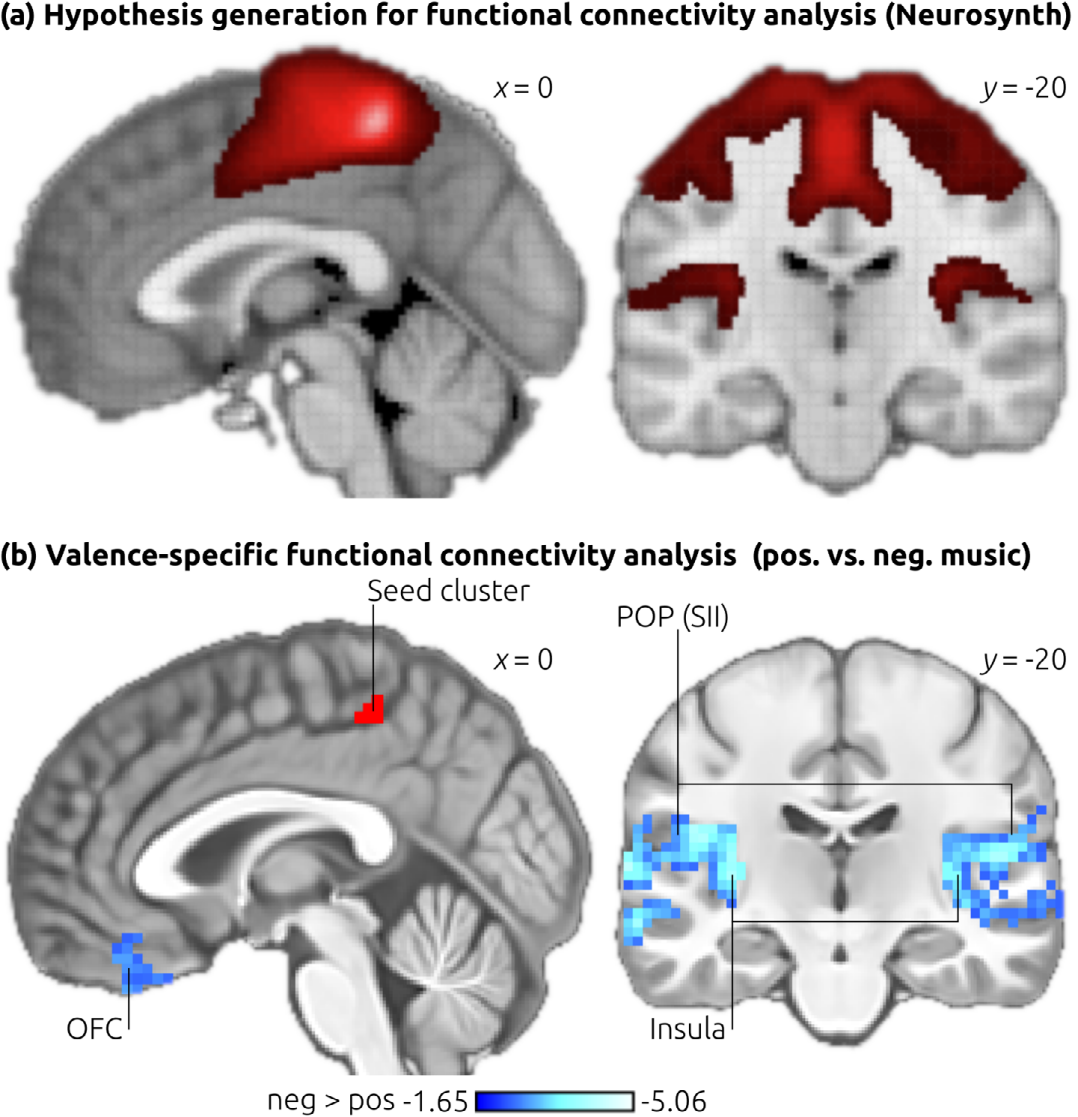
Functional connectivity analysis. To generate hypotheses for a functional connectivity analysis of area 23c, we accessed the resting‐state functional connectivity map of the peak coordinate of the ECM cluster in the posterior cingulate sulcus (area 23c) as provided by the Neurosynth platform (see main text for details). This analysis indicated functional connectivity of area 23c with MCC, lateral and medial SI, SII, and insular cortex (a). Then, a valence‐specific functional connectivity analysis was computed with the data obtained in our fMRI experiment (b), using the ECM cluster in area 23c as the seed region (illustrated by the red cluster). Results showed stronger functional connectivity of area 23c during negative compared with positive music (indicated in blue) with secondary somatosensory cortex (SII), insula, and OFC. Results are corrected for multiple comparisons (*p* < .001), using 10,000 Monte Carlo permutations. Images are shown in neurological convention, coordinates refer to MNI space, the color‐bar in (b) indicates *z*‐values. OFC, orbitofrontal cortex; POP, parietal operculum

Second, we accessed the functional connectivity map generated by Neurosynth using our peak coordinate in area 23c as a seed coordinate (Neurosynth provides voxel‐based resting‐state functional connectivity analyses based on a pool of 1000 fMRI datasets). That map is shown in Figure 4a, indicating functional connectivity of area 23c during rest with lateral and medial (primary) somatosensory cortex (SI), MCC, secondary somatosensory cortex (SII) in the parietal operculum (POP), and posterior (granular) insular cortex. (Notably, as we will discuss in more detail further below, two of these regions, namely SII and insula are among the four “core regions consistently activated by pain”; Xu et al., 2020).

Third, based on these observations, we compared the functional connectivity of area 23c with these regions (SI, SII/POP, MCC, posterior insula) between the two conditions (positive vs negative) of the present experiment, hypothesizing that area 23c would show stronger functional connectivity with these regions during negative, compared with positive, music. Results of this valence‐specific functional connectivity analysis are shown in Figure 4b. In line with our hypothesis, area 23c showed significantly stronger functional connectivity during negative compared with positive music with secondary somatosensory cortex (SII/POP) and insula. Additional regions with stronger functional connectivity during negative compared to positive music were observed in the medial orbitofrontal cortex (mOFC), auditory cortex, and the anterior hippocampal formation (for details see Table 2). No significant valence‐specific functional connectivity was observed with SI or MCC.

**TABLE 2:** Valence‐specific functional connectivity analysis

Area	Vol. (mm^3^)	*z*‐value (mean ± SD)	*z*‐value (max)	MNI‐coord.
(a) Contrast of functional connectivity maps: Negative > positive music stimuli				
L auditory cortex (core and belt regions)	29,916	−2.96 ± 0.48	−5.06	−60, −12, 7
POP			−4.44	−45, −15, 21
STGp			−4.21	−66, −21, 12
Post. insula			−4.24	−36, −18, 6
Mid insula			−4.10	−36, 0, −6
Ant. Insula			−3.81	−27, 15, −15
Post. STS			−3.29	−45, −48, 15
Ant. Hippocampus			−3.04	−27, −12, −21
R post. Insula	22,113	−2.89 ± 0.42	−4.43	42, −12, 4
Mid insula			−3.99	45, 0, 3
POP			−3.97	54, −21, 15
Post. STS			−3.70	51, −3, 6
R parahipp. cortex/collateral sulcus	4,158	−2.71 ± 0.33	−3.63	27, −45, −2
L parahipp. cortex/collateral sulcus	4,374	−2.85 ± 0.41	−3.95	−24, −45, −8
L OFC (vmPFC)	3,861	−2.60 ± 0.24	−3.53	−9, 27, −23
(b) Contrast of functional connectivity maps: Positive > negative music stimuli				
R cerebellum	6,210	2.70 ± 0.34	3.91	12, −90, −20

*Note*: The table lists the results of the functional connectivity analysis of the ECM cluster in the posterior cingulate sulcus (area 23c) as seed region (illustrated by the red cluster in Figure 4b): the results of the contrast negative > positive are listed in (a), and results of the opposite contrast are listed in (b). Results indicate stronger functional connectivity of area 23c during negative (compared with positive) music with auditory cortex, SII, insula, the hippocampal formation, and OFC. Results were corrected for multiple comparisons (*p* < .001), using 10,000 Monte Carlo permutations.

Abbreviations: ant., anterior; L, left; OFC, orbitofrontal cortex; parahipp, parahippocampal; POP, parietal operculum; post, posterior; R, right; STS, superior temporal sulcus; vmPFC, ventro‐medial prefrontal cortex.

#### 3.4.1 Replication of valence‐specific functional connectivity

Finally, we aimed at replicating the results of the valence‐specific functional connectivity analysis (as shown in Figure 4b and listed in Table 2) using the data from our previous study (Taruffi et al., 2017) with music evoking sadness or joy, also using area 23c as seed region. Using the same preprocessing and functional connectivity analysis pipeline described above for the main experiment (including the same seed region), the contrast maps of the valence‐specific functional connectivity (joy vs. sad) indicated local maxima in both left (*z* = −3.30, MNI coordinate: −42, −18, 21) and right (*z* = −2.76, MNI coordinate: 36, −21, 21) parietal operculum (with the cluster in the right POP extending into the posterior insula), as well as in the mOFC (*z* = −2.55, MNI coordinate: −6, 39, −18). In all of these structures, functional connectivity was stronger during negative than positive music (as in the results of the main experiment, cf. Table 2). Notably, in the parietal operculum, these local maxima were located in voxels adjacent to those observed in the functional connectivity data of the main experiment (and in the OFC within a 12 mm radius). Thus, across two independent experiments, area 23c exhibited stronger functional connectivity during negative than positive stimuli with SII/POP bilaterally, the right insula, and mOFC.

## 4 DISCUSSION

Our behavioral data indicate that mind‐wandering emerged during music listening, nearly as strongly as during rest. Consistent with this observation, and with the notion that the DMN is activated during mind‐wandering, the fMRI results show that several hubs of the DMN had high centrality not only during rest, but also during the music conditions. Thus, both mind‐wandering and neural activity in the DMN emerged during music listening. Importantly, the valence of thought contents during mind‐wandering was more positive during positive‐sounding music (joy & peaceful) than during negative‐sounding music (nervous & sad). This observation replicates results of two previous studies (Koelsch et al., 2019; Taruffi et al., 2017) and shows that music can systematically modulate the valence of thoughts during mind‐wandering. This enabled us to investigate neural correlates of positive and negative spontaneous, nonintentional thoughts. We identified that the centrality of the posterior cingulate sulcus (likely area 23c of the rostral PCC) differed as a function of the valence of thought content during mind‐wandering: during negative‐sounding music, the functional centrality of this region increased compared to both positive‐sounding music and rest. This observation replicates findings of our previous study (Taruffi et al., 2017), in which sad‐sounding music elicited more negative thoughts, and increased centrality of area 23c (with several voxels overlapping between that previous study and the current study). In addition, the present study provides answers to two issues that had remained unaddressed in the study by Taruffi et al. (2017): First, because in the present study the strength of mind‐wandering was balanced between positive and negative music, we can pin down the increased eigenvector centrality observed in the posterior cingulate sulcus (area 23c) to the valence of thought contents. Second, due to the inclusion of rest blocks in the present study, we can now determine that (a) thought contents during negative music were actually negative (and not just less positive than during joyful music), and that, therefore, (b) neural activity in the posterior cingulate sulcus is engaged specifically by negative stimuli (while it does not seem to differ between rest and positive stimuli).

It is interesting to note that the posterior cingulate sulcus (area 23c) appears to play a role in pain and pain regulation, as suggested by our Neurosynth meta‐analysis of the functional significance of this region. This notion is supported, for example, by an fMRI study using multivariate pattern analysis (Woo et al., 2014), in which voxels in area 23c provided decoding information for physical pain, but not for social rejection, thus suggesting that area 23c is involved in pain, rather than merely negative affect. Similarly, this region is activated by nociceptive somatosensory stimuli, but not matched nonpainful somatosensory stimuli (Mouraux, Diukova, Lee, Wise, & Iannetti, 2011), and meta‐analytic evidence indicates that this region is activated for experiencing pain oneself, but not observing others in pain (Lamm, Decety, & Singer, 2011). Notably, already the first neuroimaging study using ECM (Lohmann et al., 2010) reported higher centrality values in the posterior cingulate sulcus, including area 23c, when individuals were hungry (compared with when they were satiated). Because hunger is a negatively valenced affect with pain quality, that study (Lohmann et al., 2010) also directly supports the notion that area 23c is involved in pain. Corroborating this notion, the functional connectivity comparison of the present study showed higher functional connectivity of area 23c during negative music with SII, the insula, and mOFC. Replicating this finding, the same regions were then also identified in the analysis of our previous data (i.e., data from Taruffi et al., 2017). Both SII and insula are among the four “core regions” of pain that have recently been reported to be most robustly activated in functional neuroimaging experiments on pain (the two other regions being thalamus and MCC; Xu et al., 2020). Thus, the present data indicate that the posterior cingulate sulcus (area 23c) modulates activity in regions overlapping with the brain's putative pain network as a function of the valence of thoughts. (Note that this does not preclude that these regions are also involved in functions other than pain, including pleasure, that is, the exact opposite of pain; see also Iannetti, 2012, Wager et al., 2016, Salomons, Iannetti, Liang, & Wood, 2016.)

Beyond the posterior cingulate sulcus, centrality of the ventromedial prefrontal cortex (vmPFC), including medial orbitofrontal cortex (mOFC), was higher in our previous study during sad‐ than joyful‐sounding music (Taruffi et al., 2017). In the present study this region was not observed in the direct contrast of positive versus negative music, but was detected in the valence‐specific functional connectivity analysis which showed stronger connectivity of the posterior cingulate sulcus (area 23c) with the mOFC during negative than positive music. Our findings accord with the role of the orbitofrontal cortex (OFC) in the generation of spontaneous, nonintentional internal thoughts with emotional valence. For example, in a study by Tusche et al., 2014), activity in the mOFC predicted whether thoughts during task‐free rest periods were positive or negative. The notion of a role of the OFC in the generation of mind‐wandering thoughts with emotional valence is consistent with several previous findings implicating the OFC in rumination (Cheng et al., 2018; Cooney, Joormann, Eugène, Dennis, & Gotlib, 2010; Kühn, Vanderhasselt, De Raedt, & Gallinat, 2012; Zhou et al., 2020), including pain rumination (Kucyi et al., 2014).

Both the posterior cingulate sulcus (i.e., rostral PCC) and mOFC (part of the vmPFC) are subregions of anterior and posterior midline “hubs” of the DMN. The present results shed new light on these DMN regions: Our results indicate that neurons in the posterior cingulate sulcus become more influential during thoughts with negative valence, as reflected by increased eigenvector centrality and increased functional connectivity with SII, insula, and the mOFC. We suggest that the increased functional connectivity of the posterior cingulate sulcus with SII and insula is associated with the function of these areas for pain, and that the increased connectivity with mOFC is associated with the function of the mOFC for the generation of spontaneous thoughts with emotional valence. This interpretation of our results is consistent with findings showing that increased activity in vmPFC and the posterior cingulate sulcus during physical pain tracks depressive symptoms in fibromyalgia (López‐Solà et al., 2017), and that rumination about pain is associated with abnormal functional connectivity of the vmPFC/mOFC with the descending pain modulatory system in chronic pain patients (Kucyi et al., 2014).

The fact that thoughts generated during mind‐wandering can be positive or negative has important psychological and clinical implications: For example, while positive thoughts during mind‐wandering can be a source of inspiration (Franklin et al., 2013; McMillan, Kaufman, & Singer, 2013), ruminative negative thoughts are a characteristic aspect of the phenomenology of clinical depression (Hamilton, Farmer, Fogelman, & Gotlib, 2015; Hoffmann, Banzhaf, Kanske, Bermpohl, & Singer, 2016). Here we provide evidence dissociating these two thought modes, revealing that mind‐wandering with more negative thoughts is associated with neural activity in regions that may also be linked to the perception of pain. The (possibly causal) link between negative mind‐wandering thoughts and pain is supported by previous findings indicating that negative emotions and moods can lead to (somatic) pain or exacerbate it, including chronic pain (Wiech & Tracey, 2009).

Moreover, several studies with depressive patients support our notion of a link between the DMN, rumination, and pain: Depression and pain are often comorbid, and have common neural alterations (Demyttenaere et al., 2006; Wang et al., 2019), whereas antidepressants also impact on the neurological signature of physical pain (Wang et al., 2019). Meta‐analytic evidence indicates that rumination is linked to DMN activity, especially increased activation of vmPFC/OFC (Zhou et al., 2020), and that patients with major depressive disorder (MDD) have significantly higher OFC activation during resting state (Zhou et al., 2017). In addition, increased connectivity within the DMN (including anterior and posterior midline “hubs”) appears to be important in the pathophysiology of manifestations of depressive illness, whereas antidepressant treatment normalizes the connectivity within the DMN (Posner et al., 2013). Thus, in depressive patients, (i) increased OFC activity is associated with rumination (i.e., mind‐wandering with negative thoughts), (ii) there is increased functional connectivity within the DMN (including anterior and posterior midline “hubs”), and (iii) painful physical symptoms are common in depressive patients. Our results suggest that neurons within, and around the posterior cingulate sulcus play a crucial role in the link between ruminative thoughts and pain. This notion is supported by three meta‐analyses showing that (i) MDD patients with a history of suicidal behavior show increased activation in the posterior cingulate sulcus during emotional tasks (Li, Chen, Gong, & Jia, 2020), (ii) adults with MDD have increased activity in this region during emotional processing (Li & Wang, 2021), and (iii) activity in this region decreases after pharmacological treatment with antidepressants in depressed patients (Delaveau et al., 2011).

**FIGURE 5:**
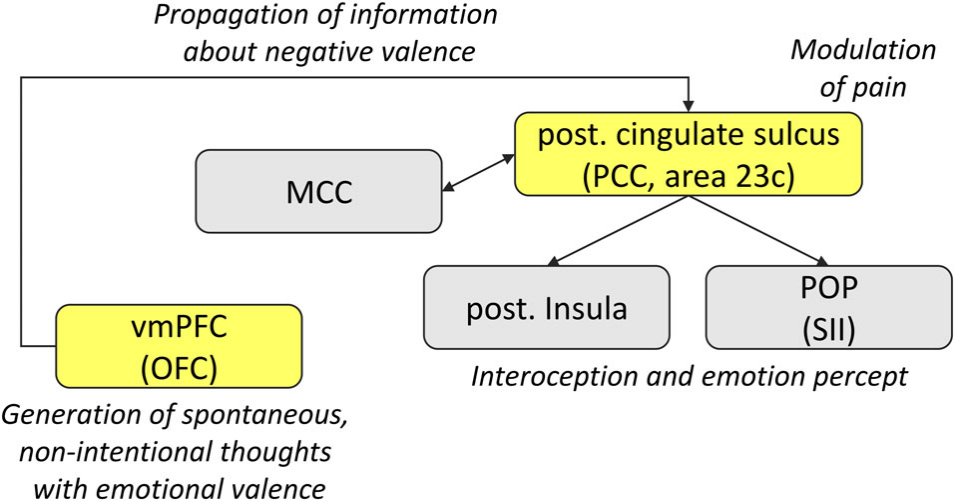
Proposed affective network underlying and mediating the painful experience of thoughts with negative valence. The structures highlighted in yellow are part of DMN midline regions. MCC, middle cingulate cortex; OFC, orbitofrontal cortex; PCC, posterior cingulate cortex; POP, parietal operculum; SII, secondary somatosensory cortex; vmPFC, ventromedial prefrontal cortex

Based on the findings discussed above, we propose a hypothetical model of a neural mechanism by which thoughts can become painful (Figure 5). The purpose of this model is to formalize specific testable hypotheses for future studies (e.g., fMRI studies using dynamic causal modeling). In this model, the OFC/vmPFC (part of the DMN) generates spontaneous, nonintentional thoughts with emotional valence. In case of negative valence, the vmPFC propagates the valence information to the posterior cingulate sulcus (area 23c of the rostral PCC, also part of the DMN) which, in turn, modulates activity in core structures of the putative pain network (SII and insula), possibly in coordination with the MCC (another core region linked to pain in prior work; Xu et al., 2020).

Our findings have important implications for the therapeutic use of music in the reduction of both rumination and pain: Our results suggest that positive‐sounding music can be used to change the valence of thoughts during rumination (i.e., mind‐wandering with repetitive negative thoughts), and thus reduce activity in the brain's putative pain system associated with negative thoughts. This assumption is also based on previous data showing that the effects of music on pain are mediated by the valence (pleasant / unpleasant) of the emotions induced (Roy, Peretz, & Rainville, 2008), in addition to a wealth of studies documenting music‐induced relief of pain for both acute and chronic pain (Cepeda, Carr, Lau, & Alvarez, 2007; Lunde, Vuust, Garza‐Villarreal, & Vase, 2019).

In addition to regions of the DMN, our eigenvector centrality mapping (ECM) indicated several cortical hubs of other resting‐state networks (RSNs), located in visual, lateral parietal, temporal, and mid‐cingulate cortex. Note that the DMN is one of several RSNs that typically appear in an independent‐component analysis (ICA) of resting‐state fMRI data (Smith et al., 2009). Other RSNs are, for example, medial and lateral visual networks (which usually appear in ICAs of resting‐state fMRI data as the first components, that is, as components explaining the largest amount of variance; Smith et al., 2009). This is consistent with our observation of large clusters with high centrality in visual areas. The ECM results showed only relatively small clusters in the fronto‐median cortex (including vmPFC and ACC), and in the posterior cingulate sulcus (area 23c of the PCC), indicating that these hubs of the DMN, despite their strong local connectivity, have only relatively weak global connectivity (or global network communication), and thus have less influential positions in RSNs than for example the medial temporal lobe subsystem of the DMN, or primary and secondary sensory networks (e.g., primary and higher‐order visual areas). This incidental finding should be specified in more detail by future studies.

### 4.1 Limitations

A potential limitation of the present study is that no phenomenological experience of pain was measured. This is relevant because structures of the pain network are also involved in other phenomena (such as pleasure; see also Iannetti, 2012, Wager et al., 2016, Salomons et al., 2016) positive. Moreover, we cannot determine a causal influence of area 23c on putative “pain” regions, and thus our results leave open the possibility that regions linked to the pain network actually regulated area 23a (and not vice versa). However, we can conclude from our data that (a) the valence of thoughts differed between experimental conditions (even though this difference may not affect pain perception), (b) the eigenvector centrality of area 23c (and thus the influence of that region in the brain) clearly differed between experimental conditions, with higher centrality emerging during negative thoughts, and (c) area 23c is functionally connected to brain structures that have repeatedly been reported with perceptual aspects of pain (even though they are also involved in other perceptual phenomena). Another limitation is that we cannot be certain that the observed neuronal effects in the posterior cingulate sulcus originated indeed from area 23c (or, perhaps in part, from the neighboring area 5 Ci). Both area 23c and area 5 Ci are relatively small, and there is considerable inter‐individual variability regarding the border between these areas (Scheperjans, Grefkes, Palomero‐Gallagher, Schleicher, & Zilles, 2005). In the eigenvector centrality maps of the music blocks (Figure 2a), however, voxels with high centrality were located within, and in the vicinity, of the cingulate sulcus, rather than extending from area 5 into the cingulate sulcus, rendering it likely that neural activity in the observed cluster in the contrast between negative and positive music (Figure 3) emerged mainly from area 23c. Future fMRI studies could also acquire DTI data of the participants, and attempt to segregate area 23c based on participants' connectivity‐based cortex parcellations.

## 5 CONCLUSIONS

Our results confirm that mind‐wandering emerges naturally during music listening (nearly as strongly as during rest), and that music has the capacity to modulate the emotional valence of spontaneous, nonintentional thoughts during mind‐wandering. The fMRI results show that several hubs of the Default Mode Network (DMN) have high centrality not only during rest, but also during music listening. According to our findings, area 23c of the posterior cingulate sulcus (a subregion of the DMN) becomes more influential during thoughts with negative valence, as indexed by an increase in eigenvector centrality. Moreover, during negative thoughts, we observed an increase of the functional connectivity of area 23c with secondary somatosensory cortex and insula (two core regions of the brain's putative pain network), as well as with the medial orbitofrontal cortex / ventromedial prefrontal cortex (mOFC/vmPFC, which is also a subregion of the DMN). This reveals that mOFC/vmPFC and the posterior cingulate sulcus (area 23c) comprise the neural basis of negative spontaneous, nonintentional thoughts, and that the interplay between these structures with core areas of the putative pain network form a neural mechanism that can make negatively valenced thoughts painful. Our findings have clinical relevance for the use of positive‐sounding music to reduce rumination and influence the valence of thoughts, thus reducing activity in the brain's pain system associated with negative thoughts.

## CONFLICT OF INTEREST

The authors declare no conflict of interest.

## Supporting information


**DATA S1**: Supporting Information

## Data Availability

The data that support the findings of this study are available from the corresponding author upon reasonable request.
