# Supplementary material for: Tormenting thoughts: The posterior cingulate sulcus of the default mode network regulates valence of thoughts and activity in the brain's pain network during music listening
Source: Hum Brain Mapp. 2021 Oct 15;43(2):773–86. doi: 10.1002/hbm.25686 (PMC8720190; doi:10.1002/hbm.25686)

# Tormenting thoughts: Neurons in the posterior cingulate sulcus of the Default Mode Network regulate valence of thoughts and activity in the brain's putative pain network during music listening

Stefan Koelsch, Jessica R. Andrews-Hanna, Stavros Skouras

**Supporting Information S1.** Illustration of eigenvector centrality.

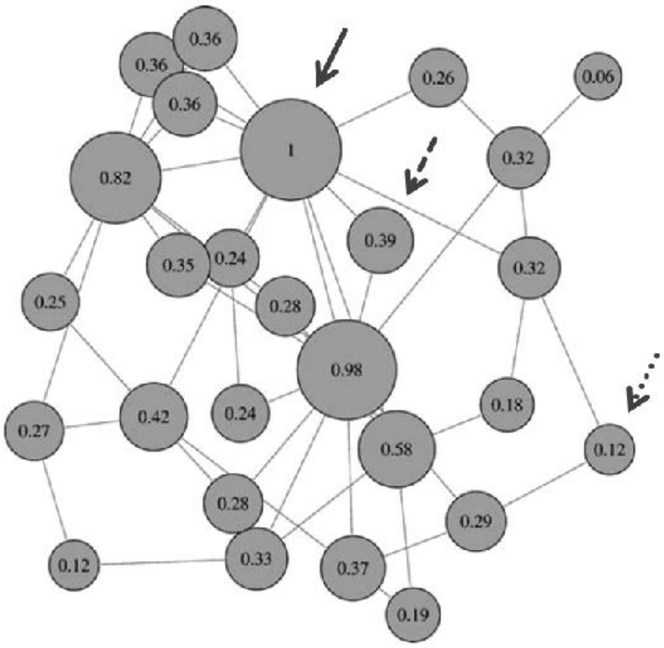


The figure illustrates a network with nodes (circles) and connections (lines). Some nodes are connected to several other nodes, whereas some nodes are only connected to two nodes, or even just one node (see the circle in the top-right corner). For each node, the eigenvector centrality value is indicated in the circle, and the circles are scaled in size according to their eigenvector centrality value. Note that eigenvector centrality does not only take the number of connections of a node into account, but also the importance of connected nodes. For example, the nodes indicated by the dashed and the dotted arrow both have two connections, but the node indicated by the dashed arrow has a higher centrality value because it is connected to the two nodes with the highest centrality values (the node with the highest centrality value is indicated by the solid arrow). ECM as applied in the current fMRI study treats each voxel as a node, and computes an eigenvector centrality value for each node (separately for each experimental condition, i.e. rest, joy, peaceful, nervous, sad), thus identifying brain regions that are influential, or important within networks of functionally interconnected structures. Formulas for the computation of eigenvector centrality are provided in: Lohmann G, Margulies DS, Horstmann A, Pleger B, Lepsien J, Goldhahn D, et al. Eigenvector Centrality Mapping for Analyzing Connectivity Patterns in fMRI Data of the Human Brain. PLoS-one. 2010;5(4):e10232. Figure published under Creative Commons Attribution License in: Koelsch, S., Skouras, S., & Lohmann, G. (2018). The auditory cortex hosts network nodes influential for emotion processing: An fMRI study on music-evoked fear and joy. PloS one, 13(1), e0190057.

**Supporting Information S2.** List of music stimuli.

**Joy stimuli**

| Title | Artist/Composer | year | genre | BPM |
|---|---|---|---|---|
| The Fire | Chris Blackwell | 2008 | Rock/Indie | 126 |
| Crackpot 3 | Terry Devine-King/Adam Drake | 2011 | Rock/Indie | 123 |
| Invigorating | Jonathan Graham Barrett | 2016 | Orchestral film-style music | 120 |
| Playtime | Owain Llwyd Brown | 2016 | Orchestral film-style music | 120 |
| 1. Symphony/I | Gustav Mahler | 1888 | Classical / Romantic (symphonic) | 104 |

**Nervous stimuli**

| Title | Artist/Composer | year | genre | BPM |
|---|---|---|---|---|
| Blackstar | Adam Drake | 2011 | Rock/Indie | 118 |
| Cinnamon Rolls 2 | Pete Masitti/John Andrew Barrow | 2012 | Rock/Indie | 120 |
| Desperation | Red Ochsenbein | 2014 | Orchestral film-style music | 110 |
| Deranged | Marie-Anne Fischer | 2018 | Orchestral film-style music | 120 |
| 1. Symphony/IV | Gustav Mahler | 1888 | Classical / Romantic (symphonic) | 120 |

**Peaceful stimuli**

| Title | Artist/Composer | year | genre | BPM |
|---|---|---|---|---|
| Blissful Dream | John Lithium | 2012 | Rock/Indie | 63 |
| Blissful Dream | Barrie Gledden | 2002 | Rock/Indie | 85 |
| Discovery | Dan Graham | 2016 | Orchestral film-style music | 60 |
| Chilled | Alexander Rudd | 2016 | Orchestral film-style music | 60 |
| 3. Symphony/III | Robert Schumann | 1850 | Classical / Romantic (symphonic) | 88 |

**Sad stimuli**

| Title | Artist/Composer | year | genre | BPM |
|---|---|---|---|---|
| In-Between | Barrie Gledden | 2002 | Rock/Indie | 72 |
| Serendipity | Francesco de Leonardis | 2002 | Rock/Indie | 90 |
| Concern | Dan Graham | 2016 | Orchestral film-style music | 60 |
| Tragic | Andrew Swarbrick | 2016 | Orchestral film-style music | 60 |
| 4. Symphony/I | Robert Schumann | 1851 | Classical / Romantic (symphonic) | 84 |

**Supporting Information S3**. Questionnaire for the assessment of emotional thoughts during mind-wandering, used in the preparatory internet experiment and the fMRI experiment.

*1) Were your thoughts completely focused on the music?*

*(Yes / No)*

*2) How much were you thinking about the music vs. something else?*

*(0 = "only about the music" ... 3 = "both" ... 6 = "only about something else")*

*3) How aware were you of where your attention was focused?*

*(0 = "completely unaware" ... 3 = "neutral" 6 = "completely aware")*

*4) Did you feel like you had control over where your thoughts went?*

*(0 = "not at all" ... 3 = "maybe" ... 6 = "very much")*

*5) Were your thoughts related to yourself?*

*(0 = "not at all" ... 3 = "a little" ... 6 = "very much")*

*6) Were your thoughts related to other people?*

*(0 = "not at all" ... 3 = "a little" ... 6 = "very much")*

*7) Was what you were thinking about rather negative or positive?*

*(-3 = "very negative" ... 0 = "neutral" ... +3 = "very positive")*

*8) Was what you were thinking about (something that would make you) rather calm or agitated?*

*(0 = "very calm" ... 3 = "neutral" ... 6 = "very agitated")*

*9) Was what you were thinking about related to the past, present or the future?*

*(-3 = "distant past" ... 0 = "present" ... +3 = "distant future" or: -9/NA = "timeless")*

*10) How relevant was what you were thinking about to the current concerns of your life?*

*(0 = "not at all relevant" ... 3 = "neutral" ... 6 = "extremely relevant")*

**Supporting Information S4:** Behavioral results of the internet experiment (*z*-standardized means and SEMs).

**(a) Valence ratings**

| **Emotion category** | **mean (*z*)** | **SEM (*z*)** |
|---|---|---|
| joy | 0.29 | 0.009 |
| peaceful | 0.42 | 0.009 |
| nervous | -0.46 | 0.009 |
| sad | -0.35 | 0.009 |

**(b) Arousal ratings**

| **Emotion category** | **mean (*z*)** | **SEM (*z*)** |
|---|---|---|
| joy | 0.36 | 0.008 |
| peaceful | -0.73 | 0.007 |
| nervous | 0.86 | 0.008 |
| sad | -0.3 | 0.008 |

**Supporting Information S5.** Behavioral results of the fMRI experiment (*z*-standardized means and SEMs)

**(a) Valence ratings**

| Emotion category | mean (*z*) | SEM (*z*) |
|---|---|---|
| joy | 0.07 | 0.18 |
| peaceful | 0.52 | 0.17 |
| nervous | -0.18 | 0.21 |
| sad | -0.32 | 0.22 |
| rest | -0.07 | 0.12 |

**(b) Arousal ratings**

| Emotion category | mean (*z*) | SEM (*z*) |
|---|---|---|
| joy | 0.09 | 0.17 |
| peaceful | -0.41 | 0.16 |
| nervous | 0.62 | 0.24 |
| sad | -0.17 | 0.17 |
| rest | -0.12 | 0.14 |

**Supporting Information S6.** ECM contrast of positive (joy & peaceful) vs. negative (nervous & sad) music, with sex, age, and framewise displacement as covariates. Results of this analysis have lower z-values than the main analysis due to considerably larger number of degrees of freedom. However, note that local maxima were observed in both area 23c and PMC (as indicated in the main analysis), and that the finding reported in the main text are not spurious results due to effects of sex, age or framewise displacement. Images are shown in neurological convention, coordinates refer to MNI space, the color-bars indicate *z*-values.

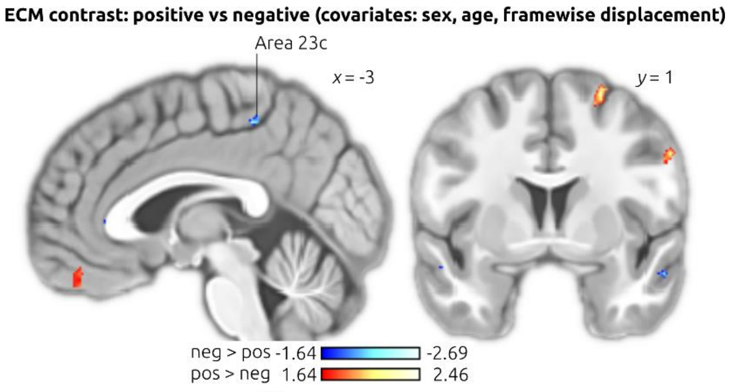

Supplement: Supplementary file 1 — DATA S1: Supporting Information [file HBM-43-773-s001.pdf]
